# Electrical Characterization of Mesh-Structured Floating-Gate Neuromorphic Transistors with Varying Mesh Sizes

**DOI:** 10.3390/mi17080967

**Published:** 2026-08-16

**Authors:** Taehwan Koo, Hyeongjin Chae, Kangmin Yoo, Hyeonseok Jeong, Juyeong Chae, Dongyeop Kim, Jineui Park, Moongyu Jang

**Affiliations:** 1 School of Nano Convergence Technology, Hallym University, Chuncheon 24252, Republic of Korea; kutae1228@gmail.com (T.K.); cogudwls3378@naver.com (H.C.); gomomo792@naver.com (K.Y.); tjr5457@naver.com (H.J.); cowndud0801@gmail.com (J.C.); kdy6380@naver.com (D.K.); park972574@gmail.com (J.P.); 2 School of Semiconductor & Display Technology, Hallym University, Chuncheon 24252, Republic of Korea; 3 Center of Nano Convergence Technology, Hallym University, Chuncheon 24252, Republic of Korea

**Keywords:** neuromorphic computing, synaptic transistor, floating gate, mesh structure, electric field concentration, perimeter-to-Area (*P*/*A*) ratio

## Abstract

This study investigates the influence of mesh-structured floating-gate (FG) geometry on the electrical and DC synaptic characteristics of flash-memory-based neuromorphic transistors. Devices with mesh sizes of 3 µm × 3 µm, 1 µm × 1 µm, 500 nm × 500 nm, and 200 nm × 200 nm were comparatively evaluated while maintaining the same channel dimensions. As the mesh size decreased, the perimeter-to-area (*P*/*A*) ratio increased from 1.33 to 20.0 µm^−1^, and the cycle-averaged memory window increased from 0.86 to 1.68 V under the same DC program/erase sequence. The 200 nm device also exhibited a read-current modulation range exceeding six orders of magnitude, compared with approximately three orders of magnitude for the 3 µm device. These trends are consistent with a greater contribution of mesh-edge regions to local electrostatic conditions and charge injection. At the same time, smaller mesh sizes produced more abrupt threshold-voltage and read-current changes during the initial program/erase steps, indicating a trade-off between response sensitivity and gradual state modulation. These results show that mesh-size scaling provides an effective geometrical design variable for tuning the memory window and readout characteristics of mesh-structured floating-gate synaptic transistors.

## 1. Introduction

With the recent advancements in the artificial intelligence (AI) industry and the explosive increase in data processing volume, the limitations of conventional computing architectures are becoming increasingly apparent [1,2,3]. As a promising solution, neuromorphic computing, which mimics the parallel processing structure of the biological brain, has emerged as a next-generation semiconductor technology capable of overcoming the von Neumann bottleneck [4,5,6,7,8,9,10,11,12,13,14]. Artificial synapses in neuromorphic systems must emulate the operational mechanisms of the biological brain to perform parallel computations through analog synaptic weight modulation. To realize this, various next-generation memory technologies, such as resistive random-access memory (RRAM), phase-change memory (PCM), and ferroelectric random-access memory (FeRAM), are actively being investigated. These devices are primarily based on a two-terminal structure, offering advantages in terms of easy array configuration and high integration density [15,16].

However, significant challenges remain for the commercialization and system-level application of two-terminal devices. For instance, RRAM suffers from severe device-to-device and cycle-to-cycle variations due to the randomness of the filament formation process, while PCM requires high programming currents for state transitions and is susceptible to thermal crosstalk [17,18]. Furthermore, FeRAM faces limitations such as the fatigue of ferroelectric materials and destructive read operations [19]. Additionally, the novel materials used in these processes exhibit poor compatibility with conventional silicon-based complementary metal-oxide-semiconductor (CMOS) processes, leading to increased manufacturing costs and difficulties in securing high yields.

In contrast, three-terminal flash-memory-based synaptic transistors provide nonvolatile charge storage and compatibility with standard CMOS processes [20]. A wide and controllable memory window is important for synaptic weight modulation. Whereas most previous studies have focused on material engineering, the effects of floating-gate (FG) geometry remain less explored. In a mesh-structured FG, reducing the mesh size increases the relative contribution of edge regions and may alter local electrostatic conditions and charge injection. However, edge-field enhancement and increased Fowler–Nordheim (F–N) tunneling should not be inferred without direct quantitative evidence.

Our previous studies demonstrated charge-storage and weight-modulation behavior in capacitors employing a mesh-type FG with the same gate stack and fundamental synaptic operation in a transistor with a 3 µm × 3 µm mesh-dot FG [21]. The discrete FG regions may help limit the lateral propagation of leakage paths associated with localized defects. However, only one mesh size was evaluated, leaving the effects of geometrical scaling unresolved.

To address this gap, this study compares transistors with mesh sizes of 3 µm × 3 µm, 1 µm × 1 µm, 500 nm × 500 nm, and 200 nm × 200 nm, fabricated using optical and electron-beam lithography. All devices use a Pt/Cr/HfO_2_/Pt/Cr/HfO_2_/SiO_2_/Si gate stack on an SOI wafer. We examine mesh-size-dependent changes in the perimeter-to-area (*P*/*A*) ratio, memory window, read-current modulation, and the sensitivity–graduality trade-off under DC program/erase conditions. The measured devices showed an increase in memory window with increasing *P*/*A* ratio. This trend is consistent with a possible contribution of edge-related electrostatic effects to charge injection, but it does not directly demonstrate local field enhancement or increased F–N tunneling. Accordingly, the principal contribution of this work is a systematic comparison of mesh-size-dependent electrical and DC synaptic characteristics rather than the definitive identification of a single mechanism.

## 2. Materials and Methods

**Figure 1:**
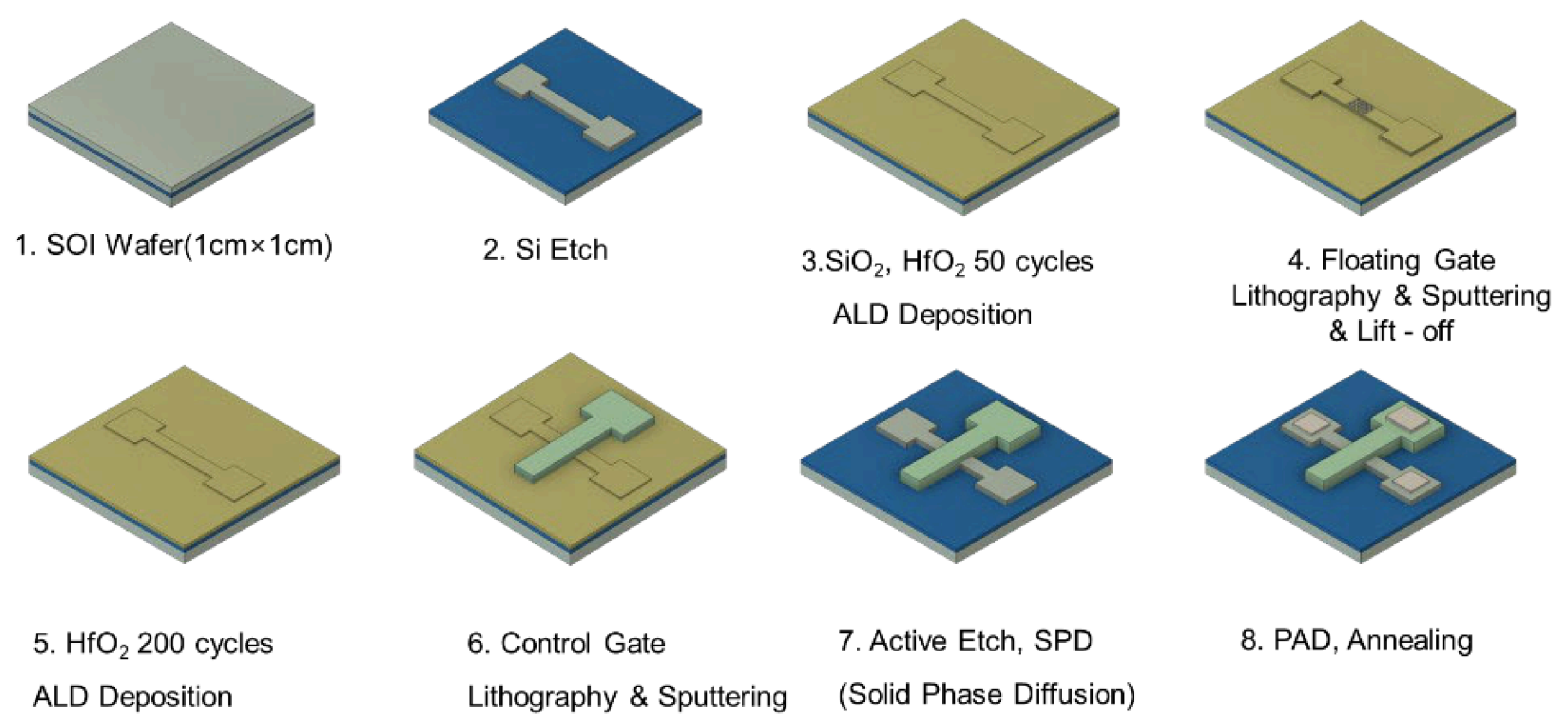
Schematic illustration of the fabrication process for the mesh-structured floating-gate neuromorphic transistor.

The neuromorphic transistor was fabricated on a p-type silicon-on-insulator (SOI) wafer with a 300 nm top-silicon layer, following the process sequence illustrated in Figure 1. The active region was defined by photolithography using a contact aligner (EVG610, EV Group, St. Florian am Inn, Austria) and reactive ion etching (RIE) using a reactive ion etcher (SHE-6T-250R, Samhan Vacuum, Paju, Republic of Korea) with CF_4_/Ar gases. An approximately 3.4 nm SiO_2_ layer was formed by dry oxidation at 900 °C using a furnace (PYRO-H 50, ULTECH, Daegu, Republic of Korea), after which a HfO_2_ layer (50 cycles) was deposited at 300 °C by atomic layer deposition (ALD) using a SPACE-S 150 system (ULTECH, Daegu, Republic of Korea) to complete the tunneling-dielectric stack [22].

A mesh-structured floating gate (FG) consisting of Pt (30 nm)/Cr (2 nm) was subsequently formed using an RF magnetron sputtering system (SHS-2M3-400T, Samhan Vacuum, Paju, Republic of Korea) and lift-off. The Cr layer was used as an adhesion layer between Pt and the underlying dielectric while being kept sufficiently thin to limit its influence on charge storage [23]. Pt was selected as the FG material because of its high work function and thermal stability during the subsequent rapid thermal annealing (RTA) process [24]. The 3 µm × 3 µm mesh pattern was defined using image-reversal photolithography, whereas the 1 µm × 1 µm, 500 nm × 500 nm, and 200 nm × 200 nm patterns were defined using electron-beam lithography (JBX-8100FS, JEOL Ltd., Akishima, Tokyo, Japan) [25].

A HfO_2_ layer (200 ALD cycles) was then deposited as the blocking dielectric, followed by formation of a Pt (150 nm)/Cr (2 nm) control-gate (CG) stack. The source and drain regions were doped using a phosphorus spin-on-doping process and activated by RTA using an SHT310R system (Samhan Vacuum, Paju, Republic of Korea) at 800 °C for 20 min.

Electrical measurements were performed at room temperature in a dark enclosure using a B1500A semiconductor device analyzer (Keysight Technologies, Santa Rosa, CA, USA). One device was evaluated for each mesh size. Transfer characteristics were recorded at *V*_DS_ = 0.1 V while *V*_GS_ was swept from −3 to 5 V in the forward direction with a 0.1 V step. All reported drain-current values were normalized by the channel width and are expressed in A/µm. Program and erase operations were performed by applying DC control-gate voltages of +5.5 and −6 V, respectively. One program/erase cycle was defined as 14 sequential measurement points, comprising seven program measurements after voltage application for 1, 10, 20, 30, 40, 50, and 60 s, followed by seven erase measurements after the same respective application times.

The threshold voltage (*V*_th_) was extracted from each transfer curve using the transconductance-extrapolation method. For the memory-window calculation, the first two cycles were excluded because they may contain initial transient instability. For each of cycles 3–5, the final programmed *V*_th_ was taken after the 60 s program step, and the final erased *V*_th_ was taken after the 60 s erase step. The three final programmed *V*_th_ values were averaged, and the three final erased *V*_th_ values were averaged separately. The memory window was then defined as the absolute difference between these two mean threshold voltages:

Δ*V*_th_ = |*V*_th,P,60s_ − *V*_th,E,60s_|

where *V*_th,P,60s_ and *V*_th,E,60s_ denote the mean final programmed and erased threshold voltages, respectively, obtained from cycles 3–5 at the 60 s endpoints. The complete five-cycle sequence therefore comprises 70 sequential measurement points, obtained from repeated program/erase measurements of the same device rather than device-to-device statistics. The subthreshold swing was extracted from the inverse slope of log_10_(*I*_d_) versus *V*_GS_ in the subthreshold region below *V*_th_, and the transconductance was calculated as d*I*_d_/d*V*_GS_.

**Figure 2:**
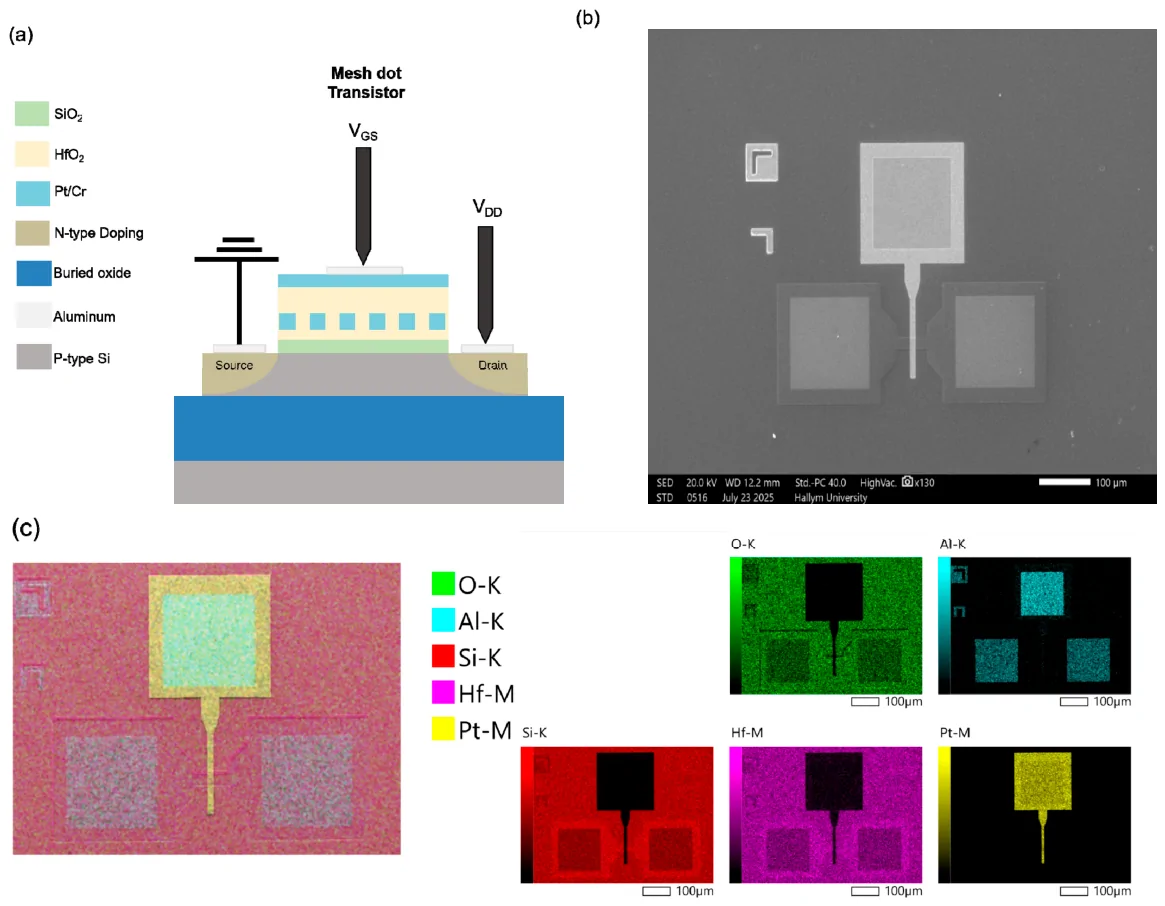
(**a**) Cross-sectional schematic of the neuromorphic transistor with a mesh-structured floating gate; (**b**) plan-view SEM image of the fabricated device; and (**c**) EDS elemental maps showing the distributions of Si, O, Al, Hf, and Pt.

Figure 2a presents a schematic representation of the vertical device stack. The plan-view scanning electron microscopy (SEM) image in Figure 2b, obtained using a scanning electron microscope equipped with energy-dispersive X-ray spectroscopy (EDS) (JSM-IT510, JEOL Ltd., Akishima, Tokyo, Japan), shows the lateral arrangement of the source/drain electrodes and gate pattern, while the EDS maps in Figure 2c show the spatial distributions of Si, O, Al, Hf, and Pt. Because these measurements are plan-view SEM and EDS analyses, they do not directly verify the individual layer thicknesses, vertical interface quality, or the absence of defects within the stacked structure. Accordingly, the layer thicknesses stated in this work are values based on the fabrication conditions.

**Figure 3:**
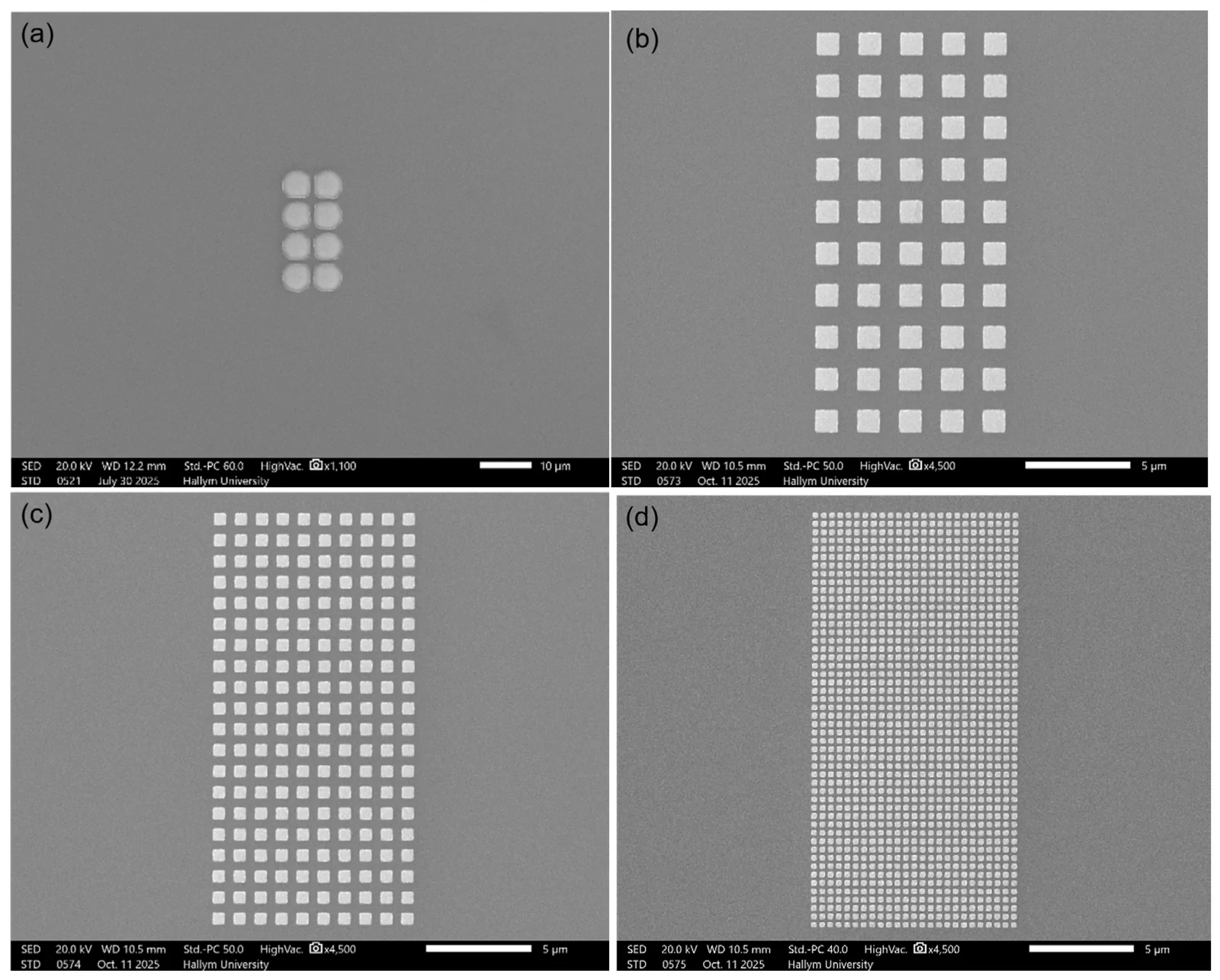
Plan-view SEM images of floating-gate arrays with mesh dot sizes of (**a**) 3 µm × 3 µm, (**b**) 1 µm × 1 µm, (**c**) 500 nm × 500 nm, and (**d**) 200 nm × 200 nm. The channel width (*W*) and length (*L*) were fixed at 20 µm and 10 µm, respectively.

To compare the mesh-size-dependent device characteristics, FG arrays with mesh-dot sizes from 3 µm × 3 µm to 200 nm × 200 nm were fabricated, as shown in Figure 3. The 3 µm × 3 µm pattern in Figure 3a was formed using image-reversal photolithography with an i-line contact aligner (365 nm) and exhibits a slightly rounded profile associated with the optical resolution. The submicron patterns in Figure 3b–d were formed using electron-beam lithography. The SEM images show laterally separated mesh dots; however, the geometrical parameters used in the following calculations were obtained from the nominal design dimensions rather than from SEM-measured dimensions.

For an array containing *N* square FG dots with side length *a*, the total FG area was calculated as *A*_total_ = *Na*^2^ and the total perimeter as *P*_total_ = *N*(4*a*). The perimeter-to-area ratio was therefore defined as *P*_total_/*A*_total_ = 4/*a*. The fill factor was calculated as the total FG area divided by the channel area of 20 µm × 10 µm (200 µm^2^). Table 1 summarizes the array configuration, number of dots, total FG area, total perimeter, *P*/*A* ratio, and fill factor. The 3 µm device differs from the submicron devices in both lithography method and total FG area; thus, the fabrication process and geometrical variables were not independently varied.

## 3. Results and Discussion

Mesh-structured floating-gate transistors with mesh sizes ranging from 3 µm × 3 µm to 200 nm × 200 nm were fabricated using optical and electron-beam lithography. The plan-view SEM images in Figure 2 and Figure 3 show the intended lateral device layout and the separation of the mesh dots, while the EDS maps show the spatial distributions of the major constituent elements. Based on these structural observations, this section discusses the measured electrical characteristics and their relationship with the geometrical parameters of the floating gate.

**Figure 4:**
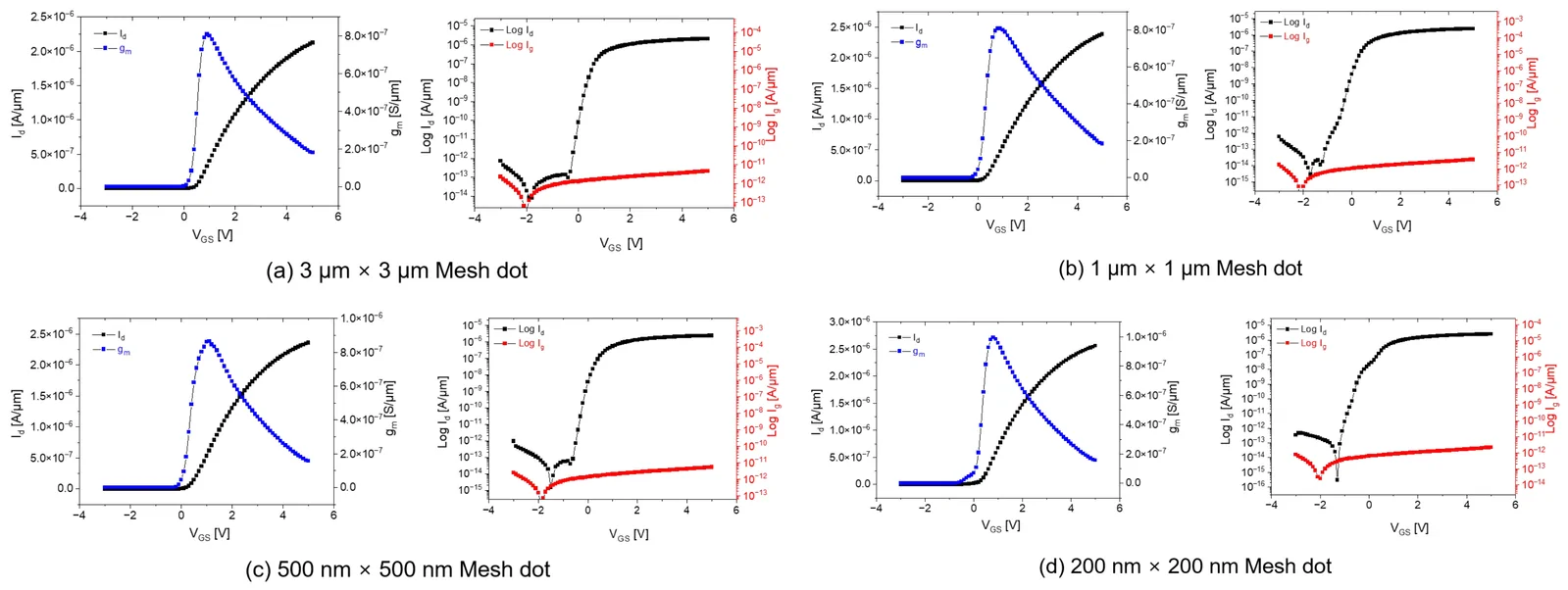
Initial transfer, transconductance, and gate leakage characteristics of the neuromorphic transistors with mesh dot sizes of (**a**) 3 µm × 3 µm, (**b**) 1 µm × 1 µm, (**c**) 500 nm × 500 nm, and (**d**) 200 nm × 200 nm.

Figure 4 compares the initial transfer characteristics, transconductance, and gate-leakage characteristics of the devices measured at *V*_DS_ = 0.1 V. All four devices exhibited gate-controlled switching with on/off current ratios exceeding 10^6^. The extracted subthreshold-swing values were 62, 104, 173, and 181 mV/dec for the 3 µm × 3 µm, 1 µm × 1 µm, 500 nm × 500 nm, and 200 nm × 200 nm devices, respectively. The corresponding peak transconductance values were 1.63 × 10^−5^, 1.68 × 10^−5^, 1.63 × 10^−5^, and 1.86 × 10^−5^ S. Thus, the peak transconductance remained within a relatively narrow range, whereas the subthreshold swing increased as the mesh size decreased. The measured gate current remained substantially lower than the drain current over the evaluated gate-voltage range, indicating that the transfer response was not dominated by direct gate leakage.

A gradual decrease in the initial *V*_th_ and a hump in the subthreshold region were observed as the mesh size decreased, with the hump being most apparent for the 200 nm × 200 nm device. These features qualitatively suggest that edge-related electrostatic modulation and locally earlier channel inversion may influence the initial turn-on behavior [26,27,28]. Process-induced variations may also contribute; therefore, the edge-related interpretation is considered a plausible explanation rather than a uniquely established mechanism.

**Figure 5:**
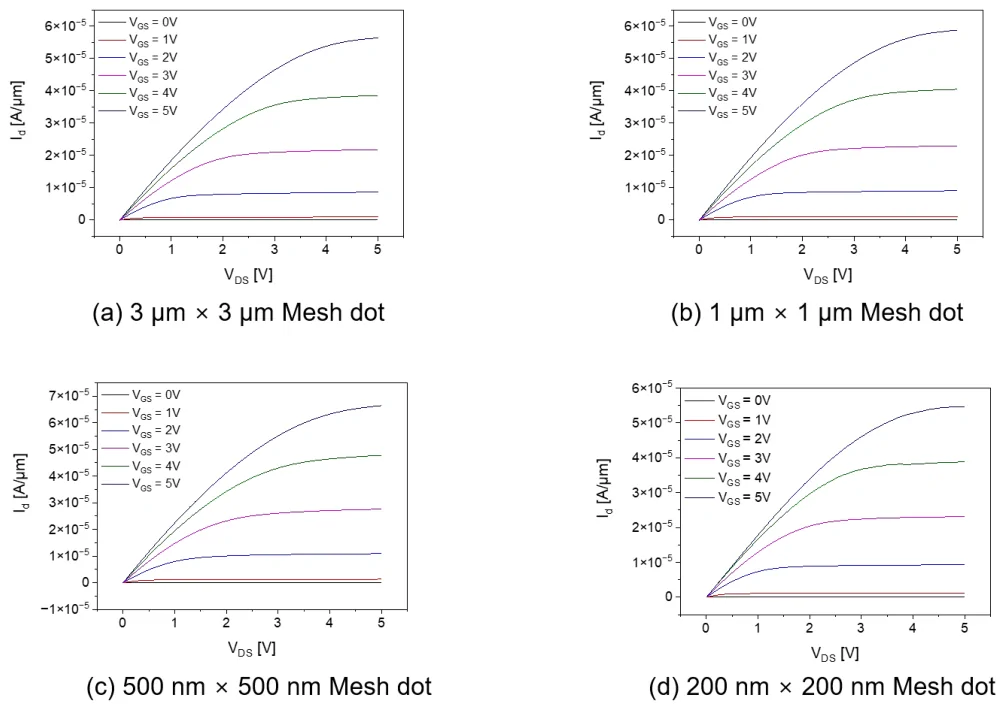
Initial output characteristics (*I*_d_–*V*_DS_) of neuromorphic transistors with mesh dot sizes of (**a**) 3 µm × 3 µm, (**b**) 1 µm × 1 µm, (**c**) 500 nm × 500 nm, and (**d**) 200 nm × 200 nm.

The on/off current ratios exceeding 10^6^ indicate that gate-controlled switching was maintained for all mesh sizes after patterning. In Figure 5, the approximately linear increase in drain current in the low-*V*_DS_ region is consistent with approximately ohmic source/drain contacts for the measured devices.

**Figure 6:**
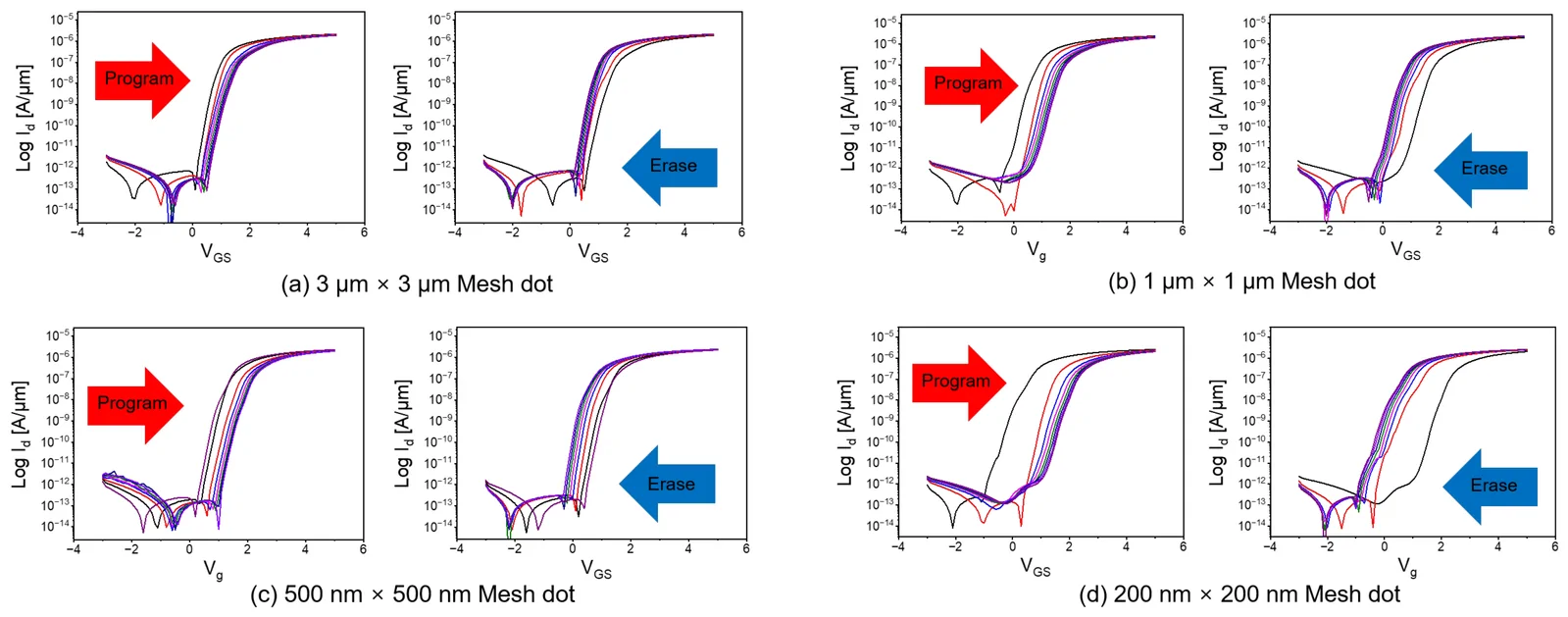
Program/erase transfer characteristics (log *I*_d_–*V*_GS_) for one cycle under +5.5 V program and −6 V erase operations: (**a**) 3 µm × 3 µm, (**b**) 1 µm × 1 µm, (**c**) 500 nm × 500 nm, and (**d**) 200 nm × 200 nm mesh devices.

Figure 6 presents the transfer curves recorded after the sequential program and erase steps within one cycle. Application of the +5.5 V program bias shifted the transfer curves toward higher gate voltage, whereas the −6 V erase bias shifted them in the opposite direction. The *V*_th_ value of each curve was extracted using the transconductance-extrapolation method described in Section 2. These extracted values were used to evaluate the *V*_th_ evolution, while the drain current at a fixed read voltage of *V*_GS_ = 1 V was used for the current-modulation analysis. The opposing transfer-curve shifts are consistent with reversible charge storage and removal and qualitatively support the expected program/erase charge-transfer behavior.

**Figure 7:**
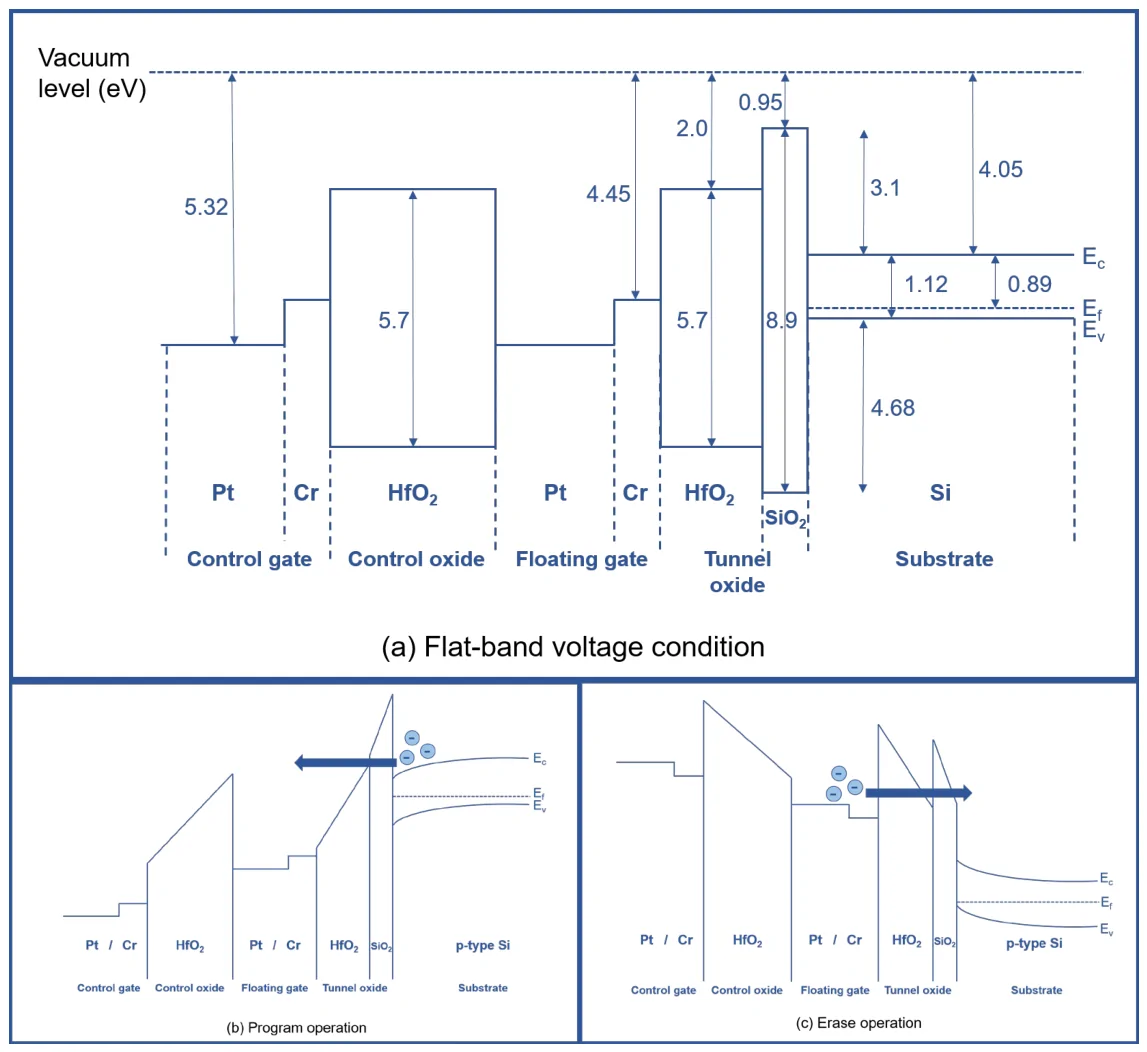
Qualitative energy-band diagrams of the Pt/Cr/HfO_2_/Pt/Cr/HfO_2_/SiO_2_/p-type Si gate stack under (**a**) flat-band, (**b**) program, and (**c**) erase conditions. The arrows in (b) and (c) indicate the direction of electron transfer during the program and erase operations, respectively.

Figure 7 schematically illustrates the gate-stack energy bands under flat-band, program, and erase conditions. Under the positive program bias, the band bending is consistent with electron injection from the p-type Si side toward the floating gate through the tunneling dielectric. Under the negative erase bias, stored electrons are released in the opposite direction toward the substrate. These charge-transfer directions are consistent with the experimentally observed program- and erase-induced *V*_th_ shifts and qualitatively support the proposed operating picture. Because the diagrams are schematic, they are used as a qualitative illustration rather than as a quantitative verification of the tunneling mechanism.

**Figure 8:**
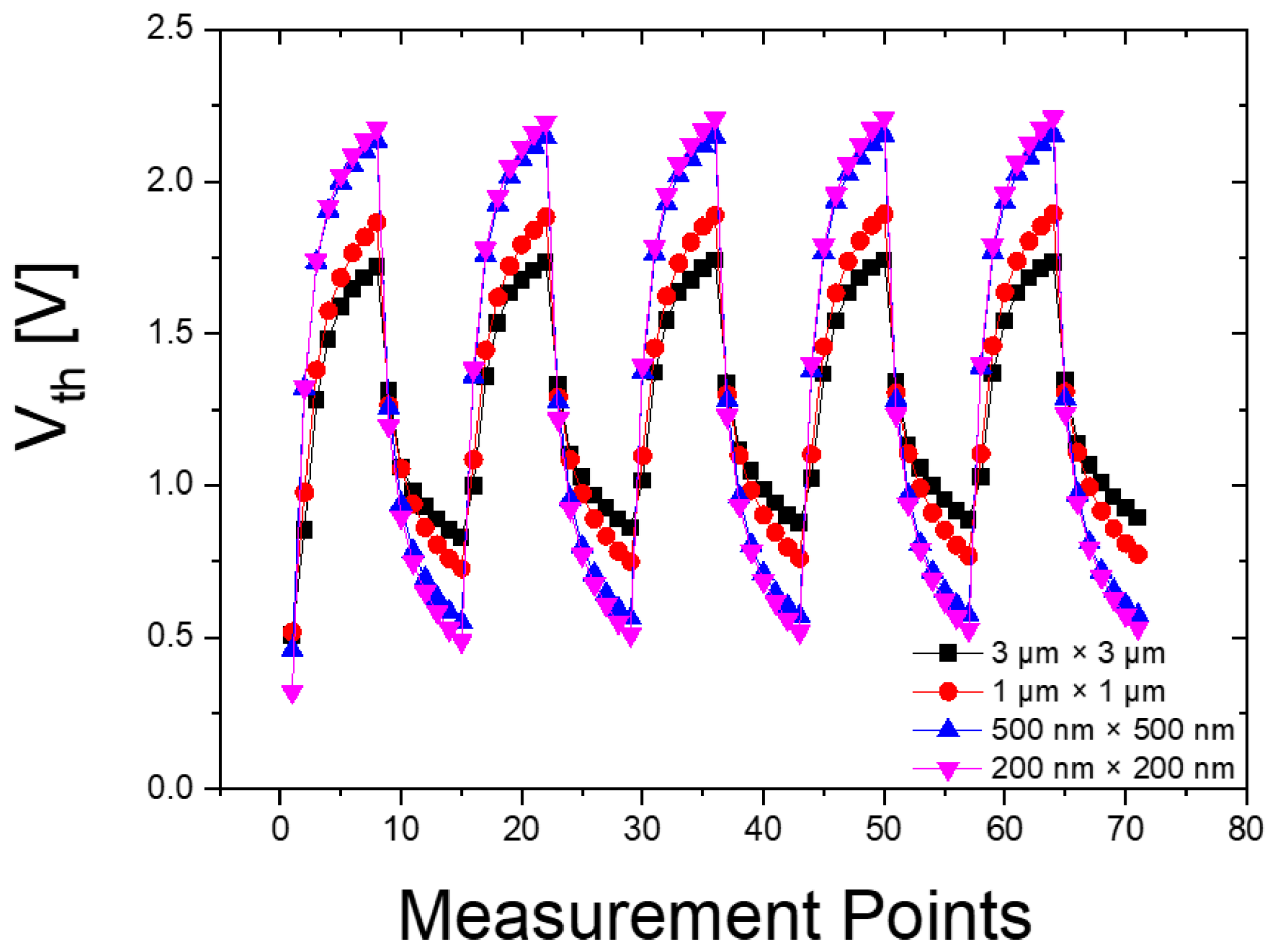
DC synaptic characteristics of devices with different mesh sizes under +5.5 V program and −6 V erase operations.

Figure 8 shows the *V*_th_ evolution over five repeated program/erase cycles. As defined in Section 2, each cycle consisted of 14 sequential measurement points: seven program measurements after voltage application for 1, 10, 20, 30, 40, 50, and 60 s, followed by seven erase measurements after the same respective application times. Therefore, the five cycles comprise 70 sequential measurement points. The first two cycles were excluded from the memory-window calculation to reduce the influence of initial transient instability. For cycles 3–5, the *V*_th_ values obtained after the 60 s program step were averaged, and the *V*_th_ values obtained after the 60 s erase step were averaged separately. The absolute difference between these two mean endpoint values yielded memory windows of 0.86, 1.13, 1.58, and 1.68 V for the 3 µm × 3 µm, 1 µm × 1 µm, 500 nm × 500 nm, and 200 nm × 200 nm devices, respectively. These values were calculated from the mean 60 s program and erase endpoint *V*_th_ values of cycles 3–5 for one measured device per mesh size.

Under the same DC bias and stress-time sequence, the memory window increased as the mesh size decreased. The 200 nm × 200 nm device exhibited comparatively steep *V*_th_ changes during the early program and erase steps, whereas the 3 µm × 3 µm device changed more gradually. This behavior qualitatively suggests a trade-off between response sensitivity and gradual state modulation under the present DC conditions. The physical origin of this trade-off may be associated with the increased relative contribution of mesh-edge regions as the mesh size decreases, which can make charge injection more responsive to the applied bias. Consequently, a larger fraction of the state change may occur during the early program/erase steps, enhancing sensitivity while reducing the graduality of incremental state modulation. The five-cycle sequence also shows repeated within-device program/erase operation over the evaluated measurement range. Further pulse-based and longer-term evaluations will be useful for assessing synaptic characteristics beyond the present DC conditions and long-term reliability.

**Figure 9:**
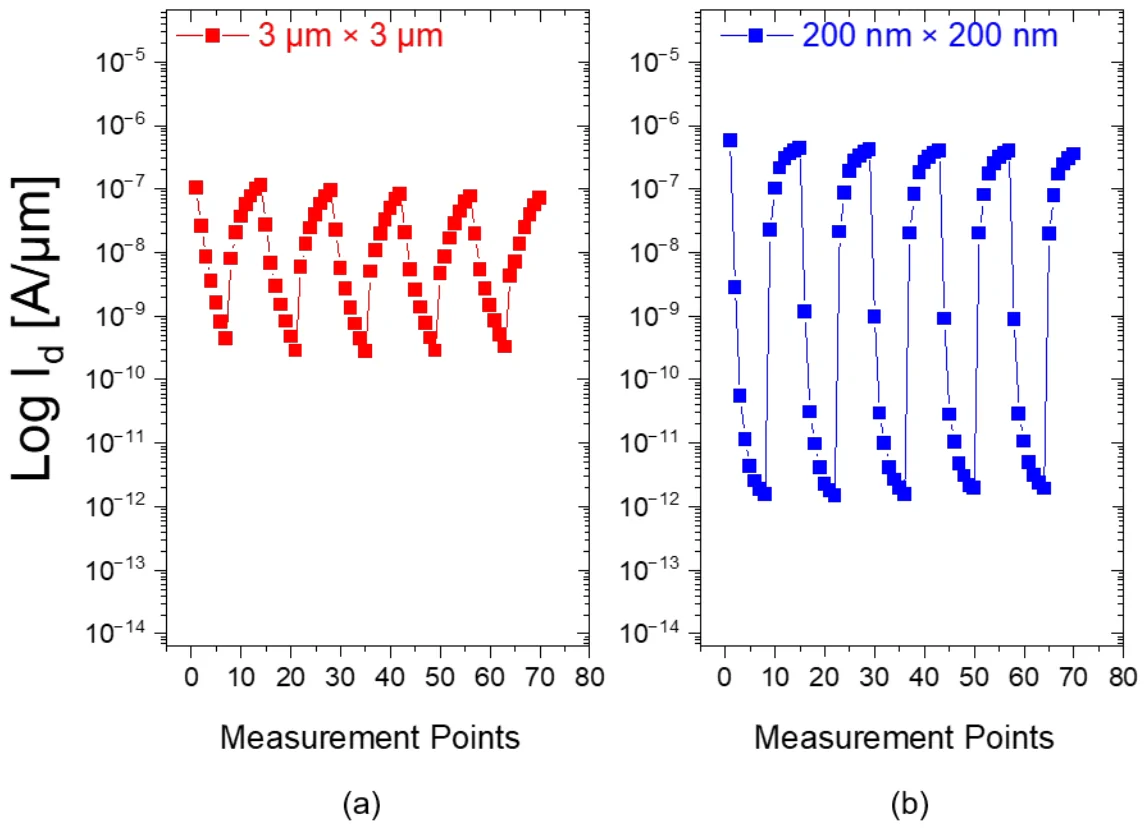
Drain-current-based DC synaptic characteristics measured at *V*_GS_ = 1 V for (**a**) the 3 µm × 3 µm mesh device and (**b**) the 200 nm × 200 nm mesh device.

To examine how the *V*_th_ modulation affects a fixed-bias readout, the drain current was evaluated at *V*_GS_ = 1 V. Figure 9 compares the log-scale current trajectories of the 3 µm × 3 µm and 200 nm × 200 nm devices during the repeated DC program/erase sequence. The 3 µm × 3 µm device showed a current-modulation range of approximately three orders of magnitude, whereas the 200 nm × 200 nm device exhibited a range exceeding six orders of magnitude, from approximately 10^−12^ to 10^−6^ A/µm. The wider range of the smaller-mesh device indicates greater read-current sensitivity, while its abrupt changes during the initial steps suggest reduced graduality of state control. Accordingly, the DC results qualitatively indicate a sensitivity–graduality trade-off associated with mesh-size scaling.

**Figure 10:**
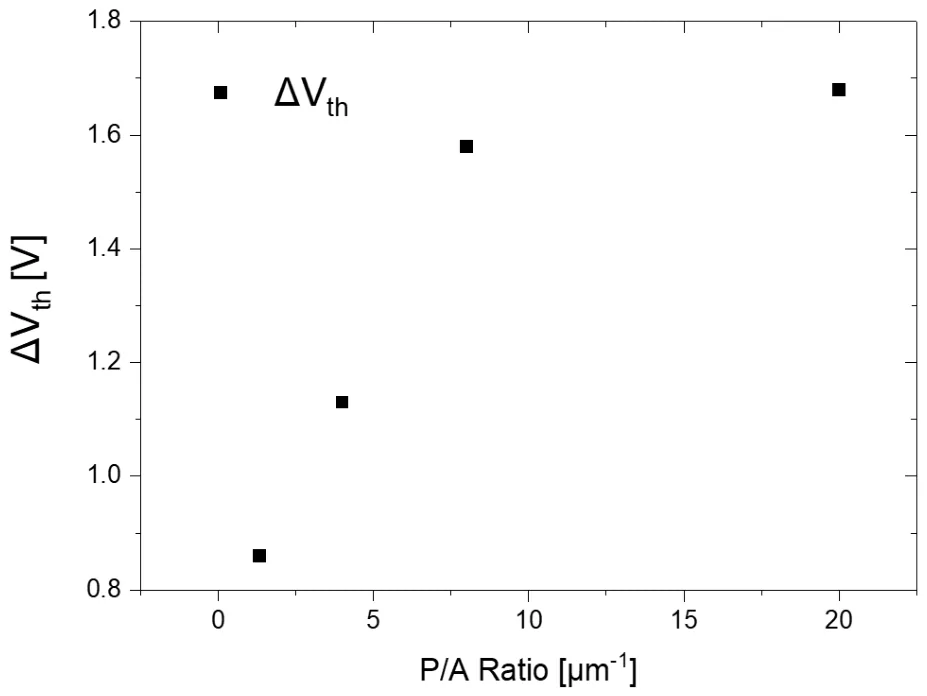
Relationship between the memory window (Δ*V*_th_) and the perimeter-to-area (*P*/*A*) ratio of the mesh-structured floating gate.

Figure 10 compares the memory window calculated from the mean 60 s program and erase endpoint threshold voltages of cycles 3–5 with the *P*/*A* ratio calculated from the design dimensions. The *P*/*A* ratio increased from 1.33 to 20.0 µm^−1^ as the mesh size decreased from 3 µm × 3 µm to 200 nm × 200 nm, while the corresponding memory window increased from 0.86 to 1.68 V. Thus, the four measured devices show a monotonic trend between the *P*/*A* ratio and the memory window. The increased relative contribution of edge regions may alter the local electrostatic conditions for charge injection, and the strong electric-field dependence of Fowler–Nordheim tunneling provides a physically plausible framework for interpreting this trend, although enhanced F–N tunneling is not directly demonstrated in the present study [29]. Because the 3 µm device also differed in lithography method, total FG area, and fill factor, the observed variation is interpreted as a geometry-associated trend rather than an effect arising solely from the *P*/*A* ratio. Overall, the results qualitatively suggest that mesh-size scaling influences charge-injection and storage behavior.

## 4. Conclusions

This study systematically compared mesh-structured floating-gate synaptic transistors with mesh-dot sizes of 3 µm × 3 µm, 1 µm × 1 µm, 500 nm × 500 nm, and 200 nm × 200 nm. Under the same DC program/erase sequence, the memory window calculated from cycles 3–5 increased from 0.86 V for the 3 µm × 3 µm device to 1.68 V for the 200 nm × 200 nm device. The smaller-mesh device also exhibited a wider read-current modulation range, exceeding six orders of magnitude, while showing more abrupt state changes during the initial program and erase steps. These results qualitatively indicate a mesh-size-dependent trade-off between response sensitivity and gradual state modulation.

The memory window increased monotonically with the perimeter-to-area ratio, suggesting that the increased relative contribution of mesh-edge regions influences the local electrostatic conditions for charge injection and storage. Because the 3 µm device also differed in lithography method, total floating-gate area, and fill factor, this relationship is interpreted as an observed geometry-associated trend rather than an effect determined solely by the *P*/*A* ratio. Because one device was evaluated for each mesh size, device-to-device reproducibility of this trend remains to be established. Overall, the comparative results demonstrate that mesh-size control is an effective design variable for tuning the DC memory and readout characteristics of mesh-structured floating-gate synaptic transistors. Future pulse-based and longer-term evaluations will be needed to further assess synaptic characteristics beyond the present DC evaluation and long-term device reliability.

## Figures and Tables

**Table 1:** Geometrical Parameters of the Mesh-Structured Floating Gates with Different Mesh Sizes.

Mesh Size	Array	Number of Dots	Total Floating-Gate Area	Total Perimeter	*P*/*A* Ratio	Fill Factor
3 µm × 3 µm	2 × 4	8	72 µm^2^	96 µm	1.33 µm^−1^	36%
1 µm × 1 µm	5 × 10	50	50 µm^2^	200 µm	4.00 µm^−1^	25%
500 nm × 500 nm	10 × 20	200	50 µm^2^	400 µm	8.00 µm^−1^	25%
200 nm × 200 nm	25 × 50	1250	50 µm^2^	1000 µm	20.0 µm^−1^	25%

## Data Availability

The data that support the findings of this study are available upon request from the authors.
